# IL-10 Modulation Increases Pyrazinamide’s Antimycobacterial Efficacy against *Mycobacterium tuberculosis* Infection in Mice

**DOI:** 10.4049/immunohorizons.2200077

**Published:** 2023-06-06

**Authors:** Varun Dwivedi, Shalini Gautam, Gillian Beamer, Paul C. Stromberg, Colwyn A. Headley, Joanne Turner

**Affiliations:** * Disease Intervention & Prevention Program, Texas Biomedical Research Institute, San Antonio, TX; † Host Pathogen Interactions Program, Texas Biomedical Research Institute, San Antonio, TX; ‡ Department of Veterinary Biosciences, College of Veterinary Medicine, The Ohio State Institute, Columbus, OH

## Abstract

Mechanisms to shorten the duration of tuberculosis (TB) treatment include new drug formulations or schedules and the development of host-directed therapies (HDTs) that better enable the host immune system to eliminate *Mycobacterium tuberculosis*. Previous studies have shown that pyrazinamide, a first-line antibiotic, can also modulate immune function, making it an attractive target for combinatorial HDT/antibiotic therapy, with the goal to accelerate clearance of *M. tuberculosis*. In this study, we assessed the value of anti–IL-10R1 as an HDT along with pyrazinamide and show that short-term anti–IL-10R1 blockade during pyrazinamide treatment enhanced the antimycobacterial efficacy of pyrazinamide, resulting in faster clearance of *M. tuberculosis* in mice. Furthermore, 45 d of pyrazinamide treatment in a functionally IL-10–deficient environment resulted in sterilizing clearance of *M. tuberculosis*. Our data suggest that short-term IL-10 blockade with standard TB drugs has the potential to improve clinical outcome by reducing the treatment duration.

## Introduction

Current combinatorial treatment for drug-sensitive tuberculosis (TB) includes at least four different antituberculous drugs lasting between 4 and 9 mo for drug-sensitive strains and much longer for drug-resistant strains (https://www.cdc.gov/tb/topic/treatment/tbdisease.htm). This extended time frame for TB treatment contributes to therapy noncompliance and significantly increases the risk of developing TB drug resistance (1–9). Shortening existing TB treatment time frames would benefit treatment compliance, curtail the development of TB drug-resistant infections, and reduce the associated treatment costs. These factors are especially important when considered in the context that a majority of TB-endemic regions are socioeconomically challenged.

The addition of a host-directed therapy (HDT) to a standard TB drug regimen, with a goal to harness the immune system to facilitate *Mycobacterium tuberculosis* clearance (10), could shorten the time frame to cure and/or reduce damage to host tissues. Current HDTs largely target inflammation and metabolism or TH1 or inflammatory cytokines (11–23) but do not necessarily account for drug-specific effects on immunity. Work by Manca et al. (24) demonstrated that pyrazinamide (PZA) reduces proinflammatory network genes in vivo in *M. tuberculosis* infection in mice, which was associated with increased expression of PPAR-γ and downregulation of NF-κB pathway genes. Although this study did not measure the effects of PZA in the absence of *M. tuberculosis* infection, PPAR-γ activation is known to mitigate macrophage proinflammatory cytokine responses in vitro, associated with increased IL-10 (25). Independently, we had observed that PZA had altered antimycobactericidal properties in IL-10 knockout (KO) mice, establishing the premise for these studies.

We hypothesized that PZA promotes IL-10 production, and that removal of IL-10–mediated suppression of proinflammatory networks would enhance the tuberculocidal activity of PZA. Our studies confirmed that PZA treatment of mice (in the absence of *M. tuberculosis* infection) reduced proinflammatory cytokines and increased IL-10 production in the lung, and we subsequently tested the influence of IL-10 on PZA activity. The absence of IL-10 (IL-10 KO mice) or anti–IL-10R1 blockade accelerated PZA-mediated clearance of *M. tuberculosis* infection in vivo in mice, resulting in complete clearance of *M. tuberculosis* in as little as 45 d. We therefore demonstrate proof of principle that anti–IL-10R1 adjunct HDT combined with a single antituberculous drug, PZA, can prevent the inhibition of proinflammatory cytokine production and accelerate the clearance of *M. tuberculosis* infection.

## Materials and Methods

### Mice

Six- to eight-week-old, specific pathogen-free male or female wild type (WT) CBA/J strain mice were purchased from The Jackson laboratory (Bar Harbor, ME) and acclimated for at least 1 wk before any experimental manipulation. CBA/J IL-10^−/−^ mice (IL-10 KO) were bred in-house (stock available at The Jackson Laboratory; strain 036145 CBA.129P2(B6)-Il10<tm1Cgn>/TrnrJ) and age and sex matched with WT mice. Mice were housed in ventilated microisolator cages and maintained with sterile water and food ad libitum. Mice were euthanized at predetermined time points by CO_2_ asphyxiation. The Ohio State University or Texas Biomedical Research Institute Institutional Laboratory Animal Care and Use Committees approved animal protocols.

### *M. tuberculosis* infection and determination of bacterial load

*M. tuberculosis* Erdman (no. 35801; ATCC) was grown in Proskauer-Beck liquid medium containing 0.05% Tween 80 to midlog phase and frozen in 1 ml aliquots at −80°C. One aliquot was used for each experimental *M. tuberculosis* infection (26). Mice were infected with a low-dose aerosol of *M. tuberculosis* Erdman using an inhalation exposure system (Glas-Col, Terre Haute, IN) calibrated to deliver 50–100 CFUs to each individual mouse (26). Organ homogenates were serially diluted and plated onto 7H11 agar plates enriched with oleic acid, albumin, dextrose, and catalase (Sigma-Aldrich, St. Louis, MO) and incubated at 37°C for 3 wk. CFUs were counted to determine the burden in each organ, and CFUs were counted to determine the burden in each organ (27).

### Ab and drug treatments

WT and IL-10 KO mice were administered PZA (15 g/l; ∼150 mg/kg/d) or isoniazid (INH; 0.1 g/l) in drinking water or by oral gavage (PZA; 150 mg/kg, dissolved in water). A total of 1 mg of anti–IL-10R1 in 100 μl saline (clone: 1B1.3A; BioXCell) was injected i.p. at treatment start followed by 0.2 mg weekly for up to 45 d. Duration and timing of treatment are described in the figure legends. A subset of mice received i.p. dexamethasone (DEX; 0.08 mg in 100 μl) for 6 consecutive days (28).

### Histology

The right caudal lung lobe was isolated from individual mice and processed as described previously (29). Sections were examined by a board-certified veterinary pathologist without prior knowledge of the experimental groups and evaluated according to the granuloma number per lung.

### Lung and spleen mononuclear cell isolation, culture, and phenotyping

Lung and spleen cell suspensions were isolated as previously described (26). For some studies, live cells were counted using a Cellometer Auto 2000 (Nexcelom Bioscience, Lawrence, MA) with acridine orange/propidium iodide stain. Mononuclear cells were cultured with medium or 5 μg/ml Con A (Sigma-Aldrich) for 24 h at 37°C, 5% CO_2_. Culture supernatants were collected and stored at −80°C. Cell culture supernatants were thawed and analyzed for TNF-α, IFN-γ, and IL-10 by custom 18-multiplex panel mouse magnetic bead Luminex assay (Mouse; R&D Systems) following the manufacturer’s instructions. Mononuclear cells were suspended in incomplete RPMI media (Sigma-Aldrich) containing 0.1% sodium azide. Surface marker staining was performed as described previously (30). Specific Abs for surface marker staining were purchased from BioLegend: PerCP anti-CD4 (clone: GK1.5), allophycocyanin/Cyanine7 anti-CD8 (clone: 53-6.7), and FITC CD69 (clone: H1.2F3). In brief, cells were blocked with mouse Fc block (clone: 2.4G2; BD Biosciences) for 10 min followed by staining with fluorescent dye–conjugated Abs specific to surface markers for 20 min at 4°C in the dark. Cells were fixed, samples were acquired using a Beckman Coulter CyAn ADP flow cytometer, and results were analyzed using FlowJo software vr. 10.5 and 10.6 (Tree Star, Ashland, OR). CD4^+^, CD8^+^, CD4^+^CD69^+^, and CD8^+^CD69^+^ cells were gated from the lymphocyte’s population.

### Statistical analysis

Statistical significance was determined using Prism vr. 7 Software (GraphPad Software, San Diego, CA). Unpaired, two-tailed Student *t* test was used for two group comparisons, and one-way or two-way ANOVA with Tukey posttest was used for more than two group comparisons. Statistical significance was reported as *p < 0.05, **p < 0.01, ***p < 0.001, or ****p < 0.0001.

## Results

### Pyrazinamide treatment modifies immune cell phenotype and function

**FIGURE 1.**
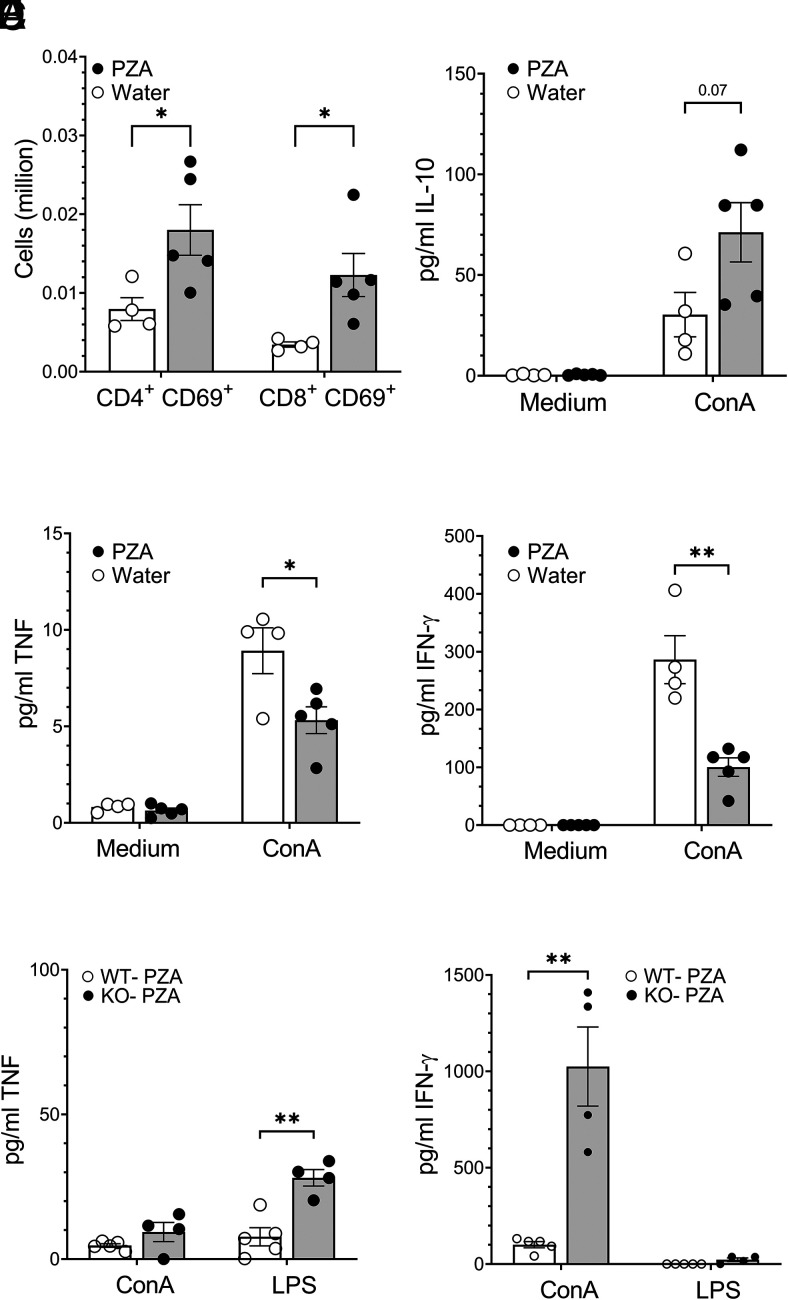
Pyrazinamide treatment modifies immune cell phenotype and function. WT mice were orally gavaged with water or PZA for 30 consecutive days. Mice were euthanized on day 30 posttreatment, and lung mononuclear cells were harvested and stained with fluorescent dye–tagged Abs specific for CD4 and CD8 in combination with CD69, acquired by flow cytometry, and analyzed by FlowJo software. Absolute numbers of CD4^+^CD69^+^ and CD8^+^CD69^+^ are shown (**A**). Lung mononuclear cells were ex vivo stimulated with medium or Con A for 24 h. Culture supernatant was analyzed for the production of IL-10 (**B**), TNF-α (**C**), or IFN-γ (**D**) by Luminex assay. WT or IL-10 KO mice were orally gavaged with water or PZA for 30 consecutive days. On day 30 posttreatment, lung mononuclear cells from WT and IL-10 KO mice were ex vivo stimulated with medium, Con-A, or LPS for 24 h. Culture supernatant was analyzed for the production of TNF-α (**E**) and IFN-γ (**F**) by Luminex assay. Basal cytokine level (medium stimulation) of each experimental group was subtracted. Data represent the mean ± SE of one of two independent experiments with three to five mice in each group. Statistical significance between WT-water and WT-PZA experimental groups was determined by Student *t* test. *p < 0.05, **p < 0.01.

To confirm that PZA has direct immunomodulatory effects in the absence of *M. tuberculosis* infection, as suggested by Manca et al. (24), we gave PZA to WT mice by oral gavage daily for 30 d and determined immune cell phenotype and function in lung and spleen homogenates. PZA treatment led to a significant increase in the number of CD4^+^ (data not shown), CD4^+^CD69^+^, and CD8^+^CD69^+^ cells in the lungs (Fig. 1A). CD69 is a classical early marker of lymphocyte activation that regulates specific functions of T cell subsets and determines the acquisition of effector or regulatory phenotypes (31). Treatment with PZA in control mice resulted in increased IL-10 and decreased TH1 cytokines in response to Con A stimulation ex vivo. Con A T cell mitogen, which triggers cross-linking of the TCR complex to activate T cells (32, 33) (Fig. 1B–D), was associated with an increase in CD4^+^CD69^+^ and CD8^+^CD69^+^ cells (Fig. 1A) (and total CD4^+^ T cells; data not shown). In contrast, treatment with LPS in lung cells of PZA-treated mice stimulated minimal IL-10 (data not shown) that was equal to controls; no differences in the absolute number of CD8^+^, CD11b^+^, CD11c^+^, and Gr1^+^ cells were observed in the lungs after PZA treatment (data not shown), and no significant increase in total cells and number of CD4^+^, CD8^+^, CD4^+^CD69^+^, CD8^+^CD69^+^, CD11b^+^, CD11c^+^, and Gr1^+^ cells was observed in the spleen (data not shown). No differences in cell number, phenotype, or function were observed in the spleen. Our data confirm that PZA treatment (in the absence of *M. tuberculosis* infection) can stimulate production of IL-10 and inhibit TH1 cytokines.

### Pyrazinamide treatment accelerates *M. tuberculosis* clearance in IL-10 KO mice

**FIGURE 2.**
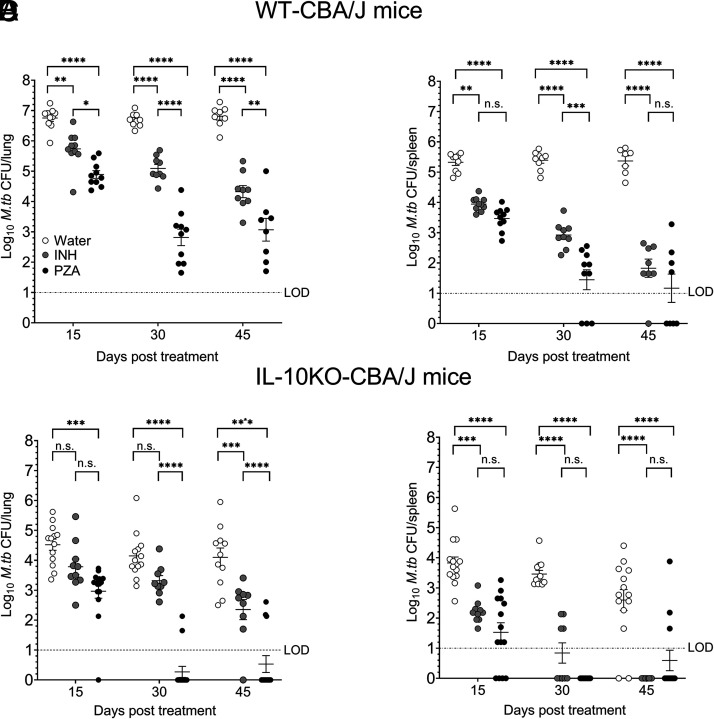
Pyrazinamide treatment accelerates *M. tuberculosis* clearance in IL-10 KO mice. WT or IL-10 KO mice were infected with *M. tuberculosis*. After 120 d, mice were treated with INH (0.1 g/l) or PZA (15g/l) in drinking water up to 45 d. *M. tuberculosis* CFUs were determined in lungs (**A** and **C**) and spleen (**B** and **D**) at days 15, 30, and 45. Data are a combined pool of two to three independent experiments, each having four to five mice in each group at all data points. Two-way ANOVA with Tukey multiple comparisons test was performed to determine statistical significance between experimental groups at each time point. *p < 0.05, **p < 0.01, ***p < 0.001, ****p < 0.0001. LOD, limit of detection.

To determine the impact of IL-10 on the efficacy of PZA, we treated *M. tuberculosis*–infected WT or IL-10 KO mice with PZA and assessed bacterial clearance. Control mice received water or INH. WT and IL-10 KO mice receiving water had an expected stable bacterial burden in their lung and spleen at all time points tested (Fig. 2A–D, open circles). In WT mice, PZA- and INH-treated mice showed a gradual decline in *M. tuberculosis* burden over time, with PZA being more efficacious in the lung (Fig. 2A, 2B). Although both INH and PZA treatment showed a significant reduction in the *M. tuberculosis* burden in lung and spleen of WT mice, both drugs failed to clear infection from the lungs within 45 d of treatment (Fig. 2A). This is consistent with previously published reports in other mouse strains (34, 35). Similar findings were observed in the spleen of WT mice, although PZA was more effective at reducing *M. tuberculosis* burden below detectable levels in several mice (Fig. 2B).

IL-10 KO mice stabilize *M. tuberculosis* at a lower burden compared with WT mice (30), as we previously observed (Fig. 2C, 2D). In IL-10 KO mice, INH and PZA treatment resulted in *M. tuberculosis* clearance from the lung and spleen (Fig. 2C, 2D), but PZA cleared *M. tuberculosis* much more effectively than INH with 80% clearance in the lung in as little as 30 d of PZA treatment. Residual *M. tuberculosis* in some IL-10 KO mice at days 30 and 45 posttreatment was likely a consequence of inefficient drug delivery because the drug was provided in the drinking water ad libitum.

**FIGURE 3.**
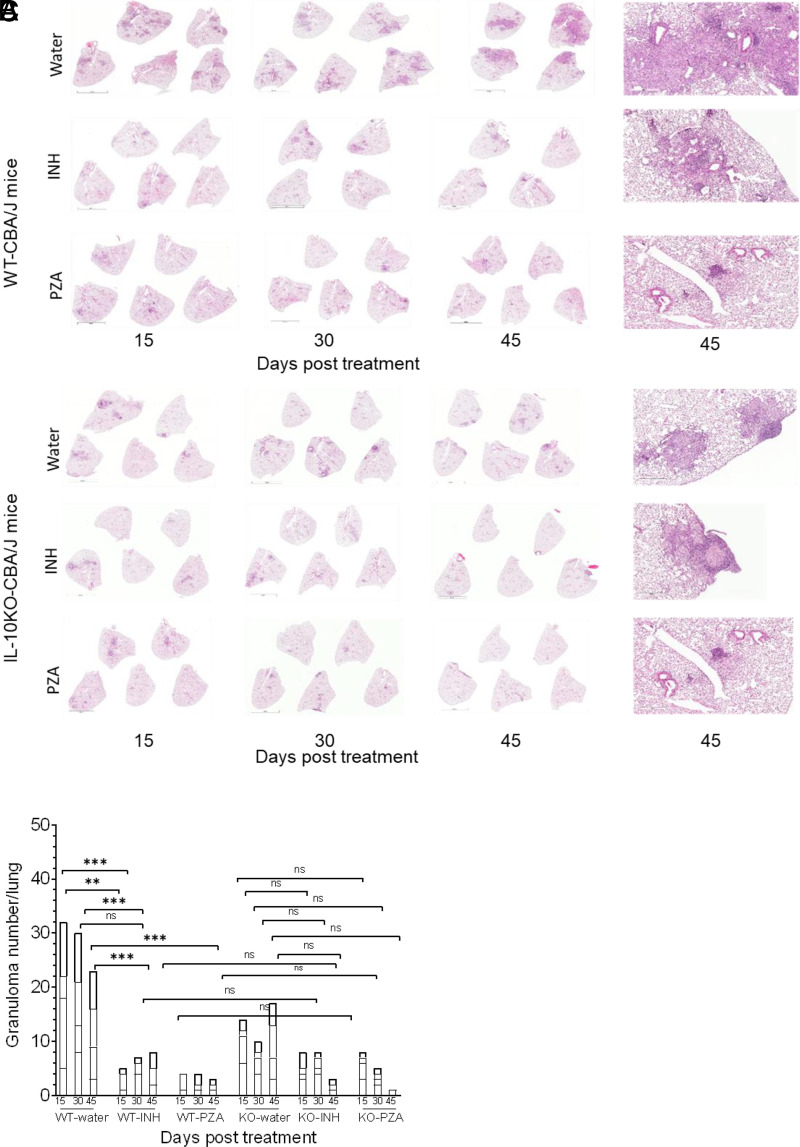
Pyrazinamide treatment reduced granuloma *M. tuberculosis* clearance in IL-10 KO mice. WT or IL-10 KO mice were infected with *M. tuberculosis*. After 120 d, mice were treated with INH (0.1g/1) or PZA (15g/l) in drinking water up to 45 d. Representative images of H&E-stained lung sections of WT and IL-10 KO mice at days 15, 30, and 45 after their respective treatment are shown to visualize tissue morphology (**A**–**C**). The higher magnification image is a representative image of day 45 tissues in each group. Each segment represents the number of granulomas in an individual mouse lung lobe. The caudal lung lobe was quantified for granuloma per lung at days 15, 30, and 45 after treatment. Two-way ANOVA with Tukey multiple comparisons test was performed to determine statistical significance between experimental groups at each time point. **p < 0.01, ***p < 0.001. Scale bars, small figures: 4 mm; large figures: 400 µm.

Analyses of lung tissues showed that WT mice receiving water had many granulomas or granuloma-like structures and had abundant cellular infiltration and lesions at day 120 (treatment start date) that remained for the duration of the study (Fig. 3A, 3C). Although there were fewer granulomas observed at day 165 postinfection because of chronic *M. tuberculosis* infection, persistent lung lesions were still observed (Fig. 3A, 3C). As anticipated, PZA and INH treatment reduced the amount of granuloma per lung in WT mice (Fig. 3A, 3C) compared with control mice. However, there was no linear reduction in granulomas despite the relative reduction in *M. tuberculosis* CFUs during the treatment regimen, as has been previously reported (36, 37). Granulomas were still visible in most mice at days 30 and 45 posttreatment for both INH and PZA. Lung tissue sections from IL-10 KO mice receiving water had fewer lung granulomas than WT mice at each time point and showed development of mature granulomas characteristic of this mouse strain (30). By day 45 of treatment with PZA or INH, the lung tissue of IL-10 KO mice contained fewer granulomas than water-treated controls, although this difference was not statistically significant (Fig. 3C). This trend was observed at all experimental time points, but statistical significance was not reached. H&E-stained lung tissue sections from WT mice are shown in Fig. 3A and from IL-10 KO mice in Fig. 3B.

Overall, our data demonstrate that the absence of IL-10 increased the antimycobacterial efficacy of PZA, resulting in improved *M. tuberculosis* clearance, and fewer granulomas, within the 45-d time frame studied.

### PZA treatment in the absence of IL-10 sterilizes *M. tuberculosis* infection

**FIGURE 4.**
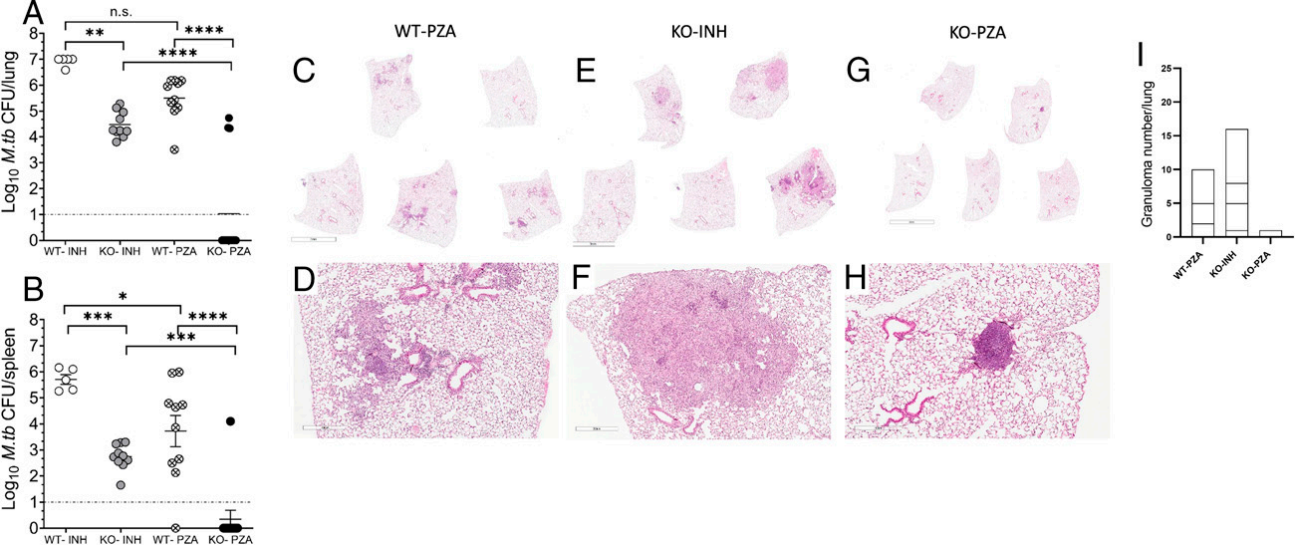
PZA treatment in the absence of IL-10 sterilizes *M. tuberculosis* infection. WT or IL-10 KO mice were infected with *M. tuberculosis*. After 120 d, mice were treated with INH (0.1g/L) or PZA (15g/l) in drinking water for 45 d. Drug treatment was then stopped and 50 d later, DEX treatment was started and continued for 6 consecutive days. Mice were euthanized 50 d after DEX treatment to determine the *M. tuberculosis* CFUs in lungs (**A**) and spleen (**B**). Data are a combined pool of one to three independent experiments, each having four to five mice in each group at all data points. One-way ANOVA with Tukey multiple comparisons test was performed to determine statistical significance between two experimental groups. *p < 0.05, **p < 0.01, ***p < 0.001, ****p < 0.0001. Representative images of H&E-stained lung sections of WT and IL-10 KO mice 50 d after DEX treatment are shown to visualize tissue morphology (**C**–**H**), and granulomas per lung was quantified (**I**). Each segment represents the number of granulomas in an individual mouse lung lobe. LOD, limit of detection. Scale bars, small figures: 3 mm; large figures: 400 µm.

PZA-treated mice were immunosuppressed with DEX to determine whether sterilization of infection could be achieved in a 45-d time frame. As anticipated, due to remaining *M. tuberculosis* in the lung at cessation of drug treatment, WT mice previously treated with INH or PZA had substantial *M. tuberculosis* burden in their lung and spleen after DEX treatment (Fig. 4A, 4B). WT mice that had been treated with INH also had a high *M. tuberculosis* burden in their lung and spleen. Although the *M. tuberculosis* burden in INH-treated IL-10 KO mice was lower than WT mice, this likely reflects the initial lower starting point (Fig. 2A, 2B). This suggests that absence of IL-10 temporarily increases the antimycobacterial activity of INH. Notably, treatment with DEX to INH-treated mice led to a substantial *M. tuberculosis* burden in their lungs, accompanied by significant cellular infiltration and lung lesions (Fig. 4A–I). In contrast, the majority of PZA-treated IL-10 KO mice had no detectable *M. tuberculosis* in the lung (10/13 mice) or spleen (12/13 mice) after DEX treatment, demonstrating a 77–92% success rate for sterilization of *M. tuberculosis* infection with PZA treatment in the total absence of IL-10. Evaluation of lung tissue (Fig. 4C–I) supported these findings, with only one mouse having a granuloma in PZA-treated IL-10 KO mice relative to three to four mice with one or more granulomas in INH-treated IL-10 KO or WT PZA-treated mice. These data indicate that PZA activity in the absence of IL-10 can resolve *M. tuberculosis* infection in as little as 45 d of treatment, and they suggest that IL-10 can, directly or indirectly, inhibit the tuberculocidal activity of PZA in vivo.

### Short-term IL-10 modulation during PZA treatment accelerates *M. tuberculosis* clearance

IL-10 is a potent anti-inflammatory immunosuppressive cytokine with a broad range of effects on innate and adaptive immunity (38). We determined that PZA treatment downregulates TH1 cytokines in the lungs (Fig. 1B–D) and spleen (data not shown) in an IL-10–dependent manner. Furthermore, PZA treatment of IL-10 KO mice resulted in significantly more TNF-α and IFN-γ in response to Con A or LPS stimulation and restored TH1 cytokines in the lungs when compared with WT-PZA mice (Fig. 1E, 1F).

**FIGURE 5.**
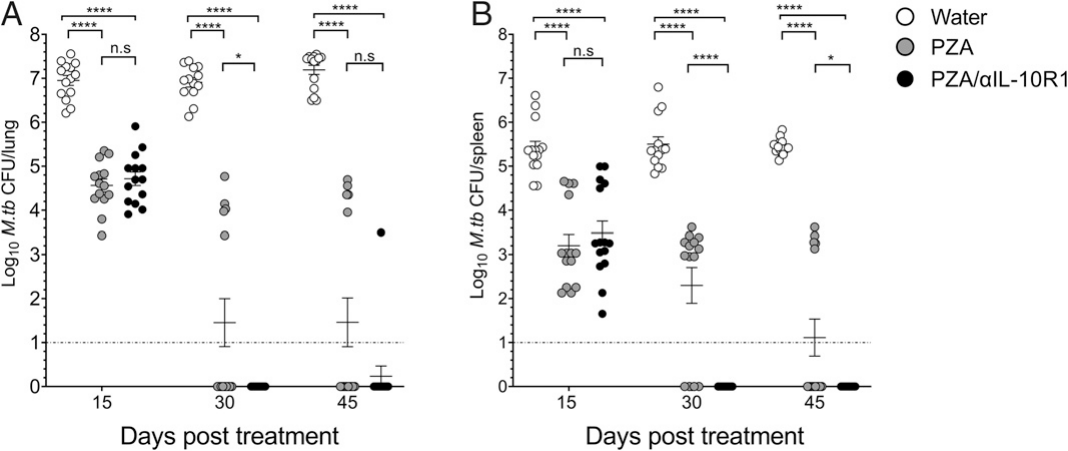
Short-term IL-10 modulation during PZA treatment accelerates *M. tuberculosis* clearance. PZA was administered to WT mice 120 d after *M. tuberculosis* infection by oral gavage once daily (**A** and **B**) for 45 consecutive days, with or without anti–IL-10R1 Ab. Mice were euthanized at days 15, 30, and 45 posttreatment, and *M. tuberculosis* burden (CFU) was determined in lung (A) and spleen (B). Data are a combined pool of three independent experiments, each having four to five mice in each group at all data points. Two-way ANOVA with Tukey multiple comparisons test was performed to determine statistical significance between experimental groups at each time point. *p < 0.05, ****p < 0.0001. LOD, limit of detection.

IL-10 deficiency is not applicable to humans; therefore, WT mice were orally gavaged with PZA to ensure uniform dosing, and we transiently blocked the IL-10R for the duration of PZA treatment in WT mice using anti–IL-10R1 Ab (Fig. 5A, 5B). PZA-treated WT mice had a significant reduction at all time points posttreatment, but no mice cleared infection below the detection limit. In contrast, 70% (7/10) of PZA/anti–IL-10R1–treated WT mice cleared *M. tuberculosis* infection below the detection limit by day 30 posttreatment, and 60% of mice (6/10) cleared *M. tuberculosis* infection below the detection limit by day 45 posttreatment (Fig. 5A, 5B). Unlike IL-10 KO mice, short-term IL-10 blockade during PZA treatment could not sterilize *M. tuberculosis* clearance, and we detected *M. tuberculosis* reactivation in all mice after DEX treatment (data not shown). Although short-term IL-10 modulation during PZA monotherapy could not achieve sterilization, inclusion of IL-10 modulation with combination TB drugs may shorten TB treatment without a risk of disease recurrence.

## Discussion

We show proof of principle that removal of IL-10 (IL-10 KO) or the blockade of its receptor interaction (anti–IL-10R1) can enhance the mycobactericidal efficacy of PZA when given as a monotherapy. This enhanced clearance of *M. tuberculosis* was not observed for INH, suggesting a specificity for PZA. PZA is known to inhibit the production of proinflammatory cytokines and stimulate IL-10 production (or IL-10 signaling pathways), both of which were observed with PZA treatment alone (Fig. 1B–D), or during *M. tuberculosis* infection (24), and were restored in the absence of IL-10 (Fig. 1E, 1F). We speculate that PZA cannot reach its full mycobactericidal potential because it also acts on the host to suppress protective TH1 and inflammatory cytokine cascades, likely through IL-10–stimulating properties. The inclusion of IL-10 inhibitors as an HDT is one strategy to improve the action of PZA, although the impact of IL-10 blockade on other TB drugs is currently unknown and should be determined. Reformulation of PZA, to reduce the IL-10–inducing properties, might be an alternate and effective approach.

The IL-10 KO mouse strain background used in these studies is a CBA/J, selected because it naturally produces abundant IL-10 in response to *M. tuberculosis* infection as has been observed in humans (39–42). We therefore used a strain that maximizes the negative impact of IL-10, and it is as yet unknown whether PZA/anti–IL-10R1 blockade would result in improved *M. tuberculosis* clearance in other mouse strains. Data from others regarding IL-10 manipulation have typically recapitulated our past findings in the CBA/J (40, 43, 44). The CBA/J IL-10 KO also presents with an additional property of forming mature granulomas as we have previously reported (30). We saw the same structures in mice treated with PZA, which confirms that the action of PZA is not inhibited by mature granuloma structure as has been demonstrated by others (45, 46). To separate the impact of mature granuloma from the direct role of IL-10, we treated WT CBA/J mice with PZA and anti–IL-10R treatment, and we found that in the absence of mature granuloma, WT mice showed an enhanced clearance of *M. tuberculosis* (Fig. 5A, 5B).

Our data provide evidence that shortening TB drug therapy for humans may be feasible, which would have significant positive implications for TB treatment. In our mouse model, the complete absence of IL-10 led to sterilizing clearance of *M. tuberculosis* in as little as 45 d, which contrasts the typically 90–120 d needed for *M. tuberculosis* clearance in mice (35, 47–51). Recent evidence suggests that the gut-lung axis may contribute to impaired lung homeostasis and play a critical role in regulating host immunity, contributing to TB development and progression (52, 53). It is feasible that microbiome differences between WT and IL-10 KO mice could account for some of our findings. However, we saw a similar trend in WT mice of the same strain background that received anti–IL-10R1 blockade, suggesting that IL-10 blockade is the main driver of enhanced protection against *M. tuberculosis* infection. This study is a proof of concept that short-term IL-10 blockade enhances PZA’s antimycobacterial activity. However, further studies need to be performed to determine the mechanism of action and the effect of short-term IL-10 blockade on combinatorial TB drug treatment regimens (2HREZ/4HR). Additional studies will be needed to determine the specific dosing regimen for anti–IL-10R1 blockade, or new reagents such as IL-10 inhibitor peptides (54) could prove to be more effective.
